# A developmentally regulated switch from stem cells to dedifferentiation for limb muscle regeneration in newts

**DOI:** 10.1038/ncomms11069

**Published:** 2016-03-30

**Authors:** Hibiki Vincent Tanaka, Nathaniel Chuen Yin Ng, Zhan Yang Yu, Martin Miguel Casco-Robles, Fumiaki Maruo, Panagiotis A. Tsonis, Chikafumi Chiba

**Affiliations:** 1 Graduate School of Life and Environmental Sciences, University of Tsukuba, Tennodai 1-1-1, Tsukuba, Ibaraki 305-8572, Japan; 2 Faculty of Life Sciences, University of Manchester, Oxford road, Manchester M13 9PT, UK; 3 Faculty of Life and Environmental Sciences, University of Tsukuba, Tennodai 1-1-1, Tsukuba, Ibaraki 305-8572, Japan; 4 Department of Biology, University of Dayton, Dayton, Ohio 45469-2320, USA

## Abstract

The newt, a urodele amphibian, is able to repeatedly regenerate its limbs throughout its lifespan, whereas other amphibians deteriorate or lose their ability to regenerate limbs after metamorphosis. It remains to be determined whether such an exceptional ability of the newt is either attributed to a strategy, which controls regeneration in larvae, or on a novel one invented by the newt after metamorphosis. Here we report that the newt switches the cellular mechanism for limb regeneration from a stem/progenitor-based mechanism (larval mode) to a dedifferentiation-based one (adult mode) as it transits beyond metamorphosis. We demonstrate that larval newts use stem/progenitor cells such as satellite cells for new muscle in a regenerated limb, whereas metamorphosed newts recruit muscle fibre cells in the stump for the same purpose. We conclude that the newt has evolved novel strategies to secure its regenerative ability of the limbs after metamorphosis.

## Abstract (web summary)

How limb regeneration in the newt is regulated at a cellular level is much debated. Here, the authors show different mechanisms acting at different developmental stages, namely stem/progenitor cells in larval regeneration and muscle fibres in the blastema regulate limb regeneration after metamorphosis.

## Graphical abstract

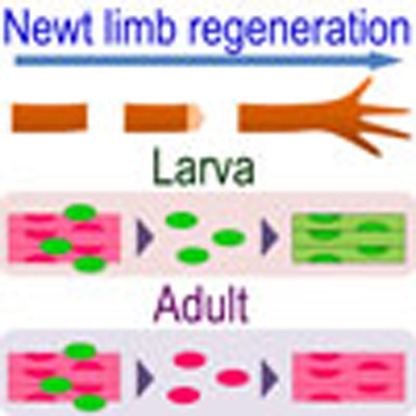

In contrast to mammals, the newt, a urodele amphibian classified into the family of *Salamandridae*, has the ability to repeatedly regenerate its limbs independently of its age. When the limb is amputated, the newt generates at the stump a cell mass called the blastema, from which a new functional limb is eventually regenerated. On the other hand, other amphibians, which also have the ability to regenerate limbs in larval stage, deteriorate or lose such ability after metamorphosis. It remains to be determined whether such an exceptional ability of limb regeneration in the newt is either attributed to a strategy, which controls regeneration in larvae, or on a novel one invented by the newt after metamorphosis^1^.

In urodeles, either skeletal muscle fibre cells (SMFCs) or muscle stem/progenitor cells (MPCs) such as satellite cells have been suggested to be contributing to new muscle in regenerated limbs. A recent study revealed that the adult newt uses SMFCs, whereas the axolotl (a neotenic species of the family *Ambystomatidae*) uses satellite cells even after artificially metamorphosed by administration of thyroid hormone, raising the argument that such a difference in the origin of muscle is attributed to differences in species^2^. However, it is still not determined whether pre-metamorphosed newt limbs employ dedifferentiation of muscle fibres or not. Although the idea of species differences makes sense, it is imperative that other questions are asked.

In this study, to address this issue, we created transgenic newts (*Cynops pyrrhogaster*) carrying a cassette, CreER^T2^<CarA-CAGGs>[EGFP]mCherry, using the I-SceI protocol^3^. In these animals, SMFCs that differentiate in the larval stage (between St 34 and St 53) are labelled by mCherry after tamoxifen administration. Using this system, we tracked SMFCs during limb regeneration in both larval and metamorphosed newts. We found that larval newts did not require muscle fibre cells to regenerate their amputated limbs. New muscle in a regenerated limb seems to originate from stem/progenitor cells, because Pax7+ cells such as satellite cells, which are muscle progenitors, are recruited to the blastema. In contrast, after metamorphosis, the muscle fibre cells in the stump were mobilized to form the blastema, eventually giving rise to new muscle in the regenerated limb. To receive a whole picture of tissue fate during limb regeneration and to compare with similar studies in axolotl, we further followed regeneration of all other tissues in adult newt via grafting experiments. We found that the principal tissues of the adult limb such as the skin, bone, muscle and nerves strictly regenerate the same type of tissues. Our current results demonstrate that the newt developmentally switches cellular mechanism of limb regeneration, revealing novel strategies that have evolved in the newt to secure its regenerative ability of the limbs after metamorphosis.

## Results

### SMFC tracking in larval newt limb regeneration

**Figure 1:**
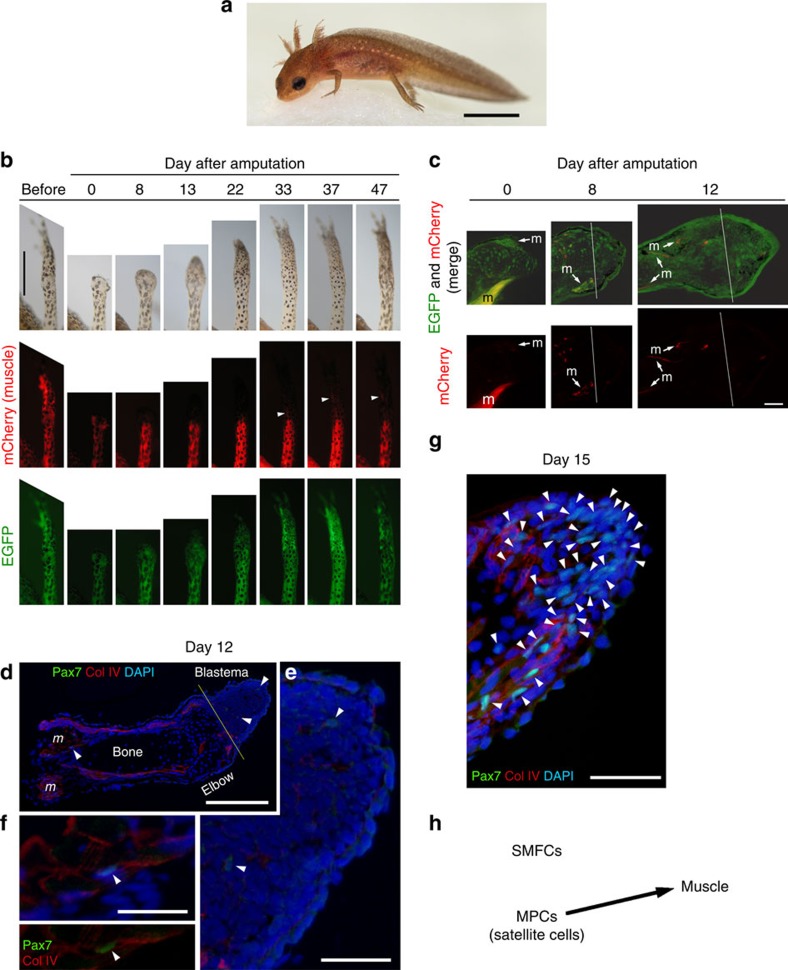
SMFC tracking in larval newt limb regeneration. (**a**) Larva (3 months old). It has four limbs, as well as the gills and tail fin. Scale bar, 4 mm. (**b**) Monitoring of SMFCs (mCherry+) during limb regeneration (*n*=6). mCherry was not detected in the regenerating part of the limb until ∼30 days when the amputated limb had almost been recovered (see Supplementary Movie 1). Arrowheads: flexor muscle for digits (see Fig. 2). Scale bar, 1 mm. (**c**) Sections of regenerating limbs (*n*=3 for each stage). SMFC-derived mCherry+ cells were not observed in the blastema. Lines: amputation site. *m*: muscle. Scale bar, 100 μm. (**d**–**f**) Pax7 immunolabelling of regenerating limbs on day 12 (*n*=3) and (**g**) day 15 (*n*=3) after amputation. (**d**) On day 12, a few Pax7+ nuclei (arrowheads) were detected in blastema cells and in satellite cells along the muscle fibres. Col IV, collagen type IV immunoreactivity. DAPI (4,6-diamidino-2-phenylindole), nuclei. Scale bar, 300 μm. The Pax7+ nuclei pointed by arrowheads were enlarged in **e** and **f**, respectively. Scale bars, 100 μm. (**g**) On day 15 when the regenerating part of the limb grew more distally, the number of Pax7+ nuclei (arrowheads) in the blastema was dramatically increased. Scale bar, 100 μm. (**h**) Summary. In larval newts, MPCs, potentially satellite cells, were recruited for new muscle during limb regeneration, whereas SMFCs were not.

In larval newts at St 56–57 (∼3 months old; Fig. 1a), whose forelimbs had grown with four digits, we found that SMFCs did not contribute to restoration of the missing part of the forelimb after amputation. When mCherry was monitored in living animals, it did not appear in the regenerating part of the limb (neither as cells from fragmented fibres nor as fibres) until ∼30 days when the amputated limb structure had almost been restored (Fig. 1b,c and Supplementary Movie 1). We subsequently examined satellite cells. We carried out immunohistochemistry with Pax7 antibody (a marker of MPCs including satellite cells)^2^ and found that Pax7-immunoreactive (Pax7+) mononucleated cells appeared in the blastema by day 12 post amputation (Fig. 1d–f; 3–17 cells per blastema, *n*=3) and increased in number in the next few days (Fig. 1g; 55–144 cells per blastema, *n*=3), suggesting that MPCs (potentially satellite cells) were recruited for new muscle during limb regeneration. Thus, these results suggest that SMFCs were not the primary source of new muscle in larval newts. Consequently, they also revealed close similarity between the larval newt and the axolotl, at least in terms of muscle regeneration (Fig. 1h).

### Appearance of mCherry in late limb regeneration

**Figure 2:**
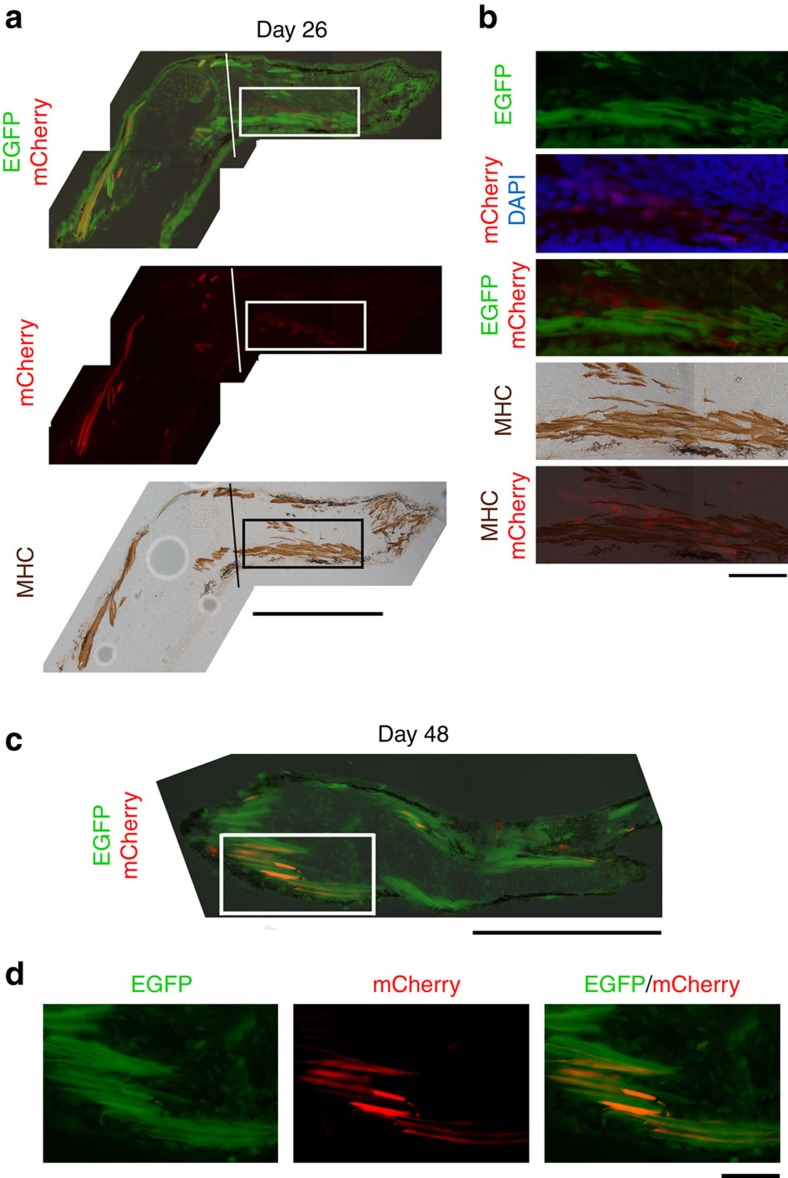
Appearance of mCherry in a late stage of larval limb regeneration. (**a**) A section of the limb on day 26 after amputation (*n*=3). mCherry was detected in mononucleated cells, which had occupied the same area as the differentiating flexor muscle for digits (rectangle). Lines: amputation site. MHC, myosin heavy chain immunoreactivity. Scale bar, 500 μm. (**b**) Enlargement of the area enclosed by the rectangle in **a**. At this stage, mCherry did not overlap with the flexor muscle. Scale bar, 100 μm. (**c**) A section of the forearm on day 48 after amputation (*n*=3). Scale bar, 500 μm. (**d**) Enlargement of the area enclosed by a rectangle in **c**. The flexor muscle for digits at this stage exhibited mCherry. Scale bar, 100 μm.

However, as differentiation of digits started taking place, an mCherry+ area became detectable along the ventral side of radius/ulna (Table 1). This area was characterized by the presence of mononucleated mCherry+ cells (they were Pax7−; Supplementary Fig. 1). Such a population was never observed in the body of animals after tamoxifen treatment (*n*=40). By day 26, these cells occupied the same area as the differentiating flexor muscle for digits. It must be noted here that at this stage there seems to be no overlap in mCherry and the flexor muscle. Therefore, we believe that these mononuclated cells did not give rise to the flexor muscle (Fig. 2a,b). The identity of these cells is not known, but a possibility of mononucleated SMFCs is excluded based on our results of mCherry monitoring throughout the period of limb regeneration (Table 1 and Supplementary Movie 1). We would like to propose that they might be fibroblasts. Our conviction stems from the fact that during axolotl muscle-less limb regeneration the tendons that transduce the action of flexor muscle to the digits are generated, and that condensations of dermal fibroblast are accumulated within the area where normally the flexor muscle would be^4^. Interestingly, the fibroblast condensations are in an oblique orientation to the flexor muscle, very similar to the one observed in our sections as well. However, by day 48 mCherry+ mononucleated cells were not anymore present, but instead the fibres of the flexor muscle were now positive for mCherry (Fig. 2c,d). This surprising result indicates that at a later stage either these mononucleated cells were differentiated to muscle fibres or that they fused with the fibres. At any rate, our observations clearly indicate that not all muscles derive from one source in newt larvae limb regeneration. Our results also suggest that these fibroblast-like cells activate the cardiac actin promoter as well, and undergo recombination in the absence of tamoxifen. The mechanism underlying this tamoxifen-independent recombination is not known, but it is known even in mammals that inducible Cre may spontaneously be activated by endogenous steroid hormones^5^. Importantly, such a mechanism seems to be elicited specifically in the fibroblast-like cells, because flexor muscle fibres, which have differentiated before the fibroblast-like cells become mCherry+, do not express mCherry.

### SMFC tracking in metamorphosed newt limb regeneration

**Figure 3:**
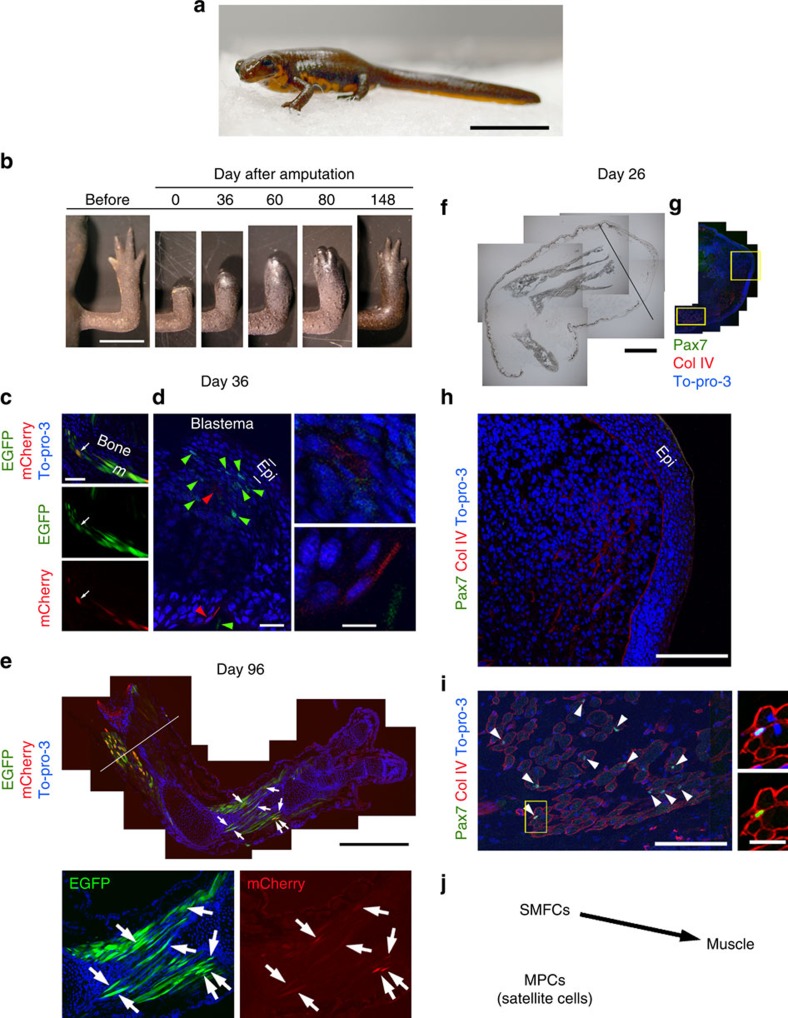
SMFC tracking in metamorphosed newt limb regeneration. (**a**) Juvenile (16 months old). Scale bar, 15 mm. (**b**) Limb regeneration. Scale bar, 5 mm. (**c**–**e**) Tracking of SMFCs (mCherry+) (*n*=2). This animal was a mosaic expressing EGFP in muscle only. mCherry+ fibres in the forearm were ∼25% of total EGFP+ fibres. (**c**) On day 36 after amputation, fragments of muscle fibres (arrows) were observed in distal regions adjacent to the blastema. Scale bar, 100 μm. (**d**) mCherry+ mononucleated cells (red arrowheads; enlarged in right-hand panels) and EGFP+ cells (green arrowheads) were observed in the blastema. Epi, epidermis. To-pro-3: nuclei. Scale bars, 50 μm (left), 10 μm (right-hand panels). (**e**) In the same limb, at day 96 after the second amputation in the upper arm (line), mCherry (arrows) and EGFP were observed only in muscle fibres. Scale bars, 1 mm (upper panel), 500 μm (lower panels). (**f**–**i**) Pax7 immunolabelling of a regenerating limb on day 26 after amputation (*n*=4). Pax7 immunoreactivity was not detected in the blastema. (**f**) Translucent image. Line: amputation site. (**g**) Merged fluorescence image. Col IV, collagen type IV immunoreactivity. To-pro-3: nuclei. Scale bar, 1 mm. (**h**) Enlargement of a region in the blastema and (**i**) a region proximal to the amputation site, enclosed by boxes in **g**. Scale bars, 250 μm. Arrowheads in (**i**) Pax7+ nuclei. An example satellite cell (box) is enlarged in the right-hand panels (upper: Col VI/To-pro-3; lower: Col IV/Pax7). Scale bar, 50 μm. (**j**) Summary. In metamorphosed newts, SMFCs were recruited for new muscle during limb regeneration, whereas MPCs such as satellite cells were not.

We performed the same experiments in newts after they metamorphosed (∼16 months old or ∼10 months after metamorphosis) (Fig. 3a,b). However, in these experiments, to preclude a possibility of contamination of other kinds of cells (such as those similar to the origin of fibroblast-like cells in larval stages), we adopted mosaic animals in which enhanced green fluorescent protein (EGFP)/mCherry expression is restricted to SMFCs. In addition, we carried out a first amputation in the middle of the forearm, and after the blastema has been formed (30–40 days after amputation) we carried out a second amputation in the middle of the upper arm of the same limb to collect the blastema for analysis. We then kept these animals for longer than 3 months, to examine later stages of regenerating limbs. Amputation on the upper arm ensures discontinuity between muscles in the regenerated forearm and those in the stump of the upper arm (see Methods).

We found that SMFCs were mobilized to regenerate new muscle (Fig. 3c–e). In this stage, the ratio of muscle fibres expressing mCherry to all muscle fibres in the forelimb was low (21–26%, *n*=4). Histological examination revealed that fragments of muscle fibres appeared near the stump, and that mononucleated cells expressing mCherry were present in the blastema (Day 36, *n*=2; Fig. 3c,d; also see Supplementary Fig. 2 and Supplementary Movie 2, which shows a three-dimensional image of fragments from EGFP+ fibres near the stump and EGFP+ mononucleated cells in the blastema in another section of the same regenerating limb). Importantly, a number of mCherry+ SMFCs were found in regenerated muscle (Day 96, *n*=2, Fig. 3e), suggesting that SMFCs in the stump dedifferentiated to form the blastema and redifferentiated to the SMFCs themselves. It must be noted that the mCherry signal was not detected in other types of cells. Moreover, the muscle in the stump, which could be tracked by EGFP, gave rise to only the regenerated muscle, but not to other tissues. Next, we examined whether satellite cells participate in limb regeneration. Pax7 immunoreactivity was not detected in the blastema even though Pax7+ satellite cells were clearly observed along SMFCs in a more proximal part to the amputation plane (Fig. 3f–i). Consequently, our results suggest that the origin of muscle changed from stem/progenitor cells to SMFCs in the newt as development proceeded beyond metamorphosis (Fig. 3j). This result is different from the one received when axolotls were forced to metamorphose^2^. In that experiment, SMFCs did not contribute to regenerated muscle.

### Lineage trace in adult limb regeneration by transplantation

**Figure 4:**
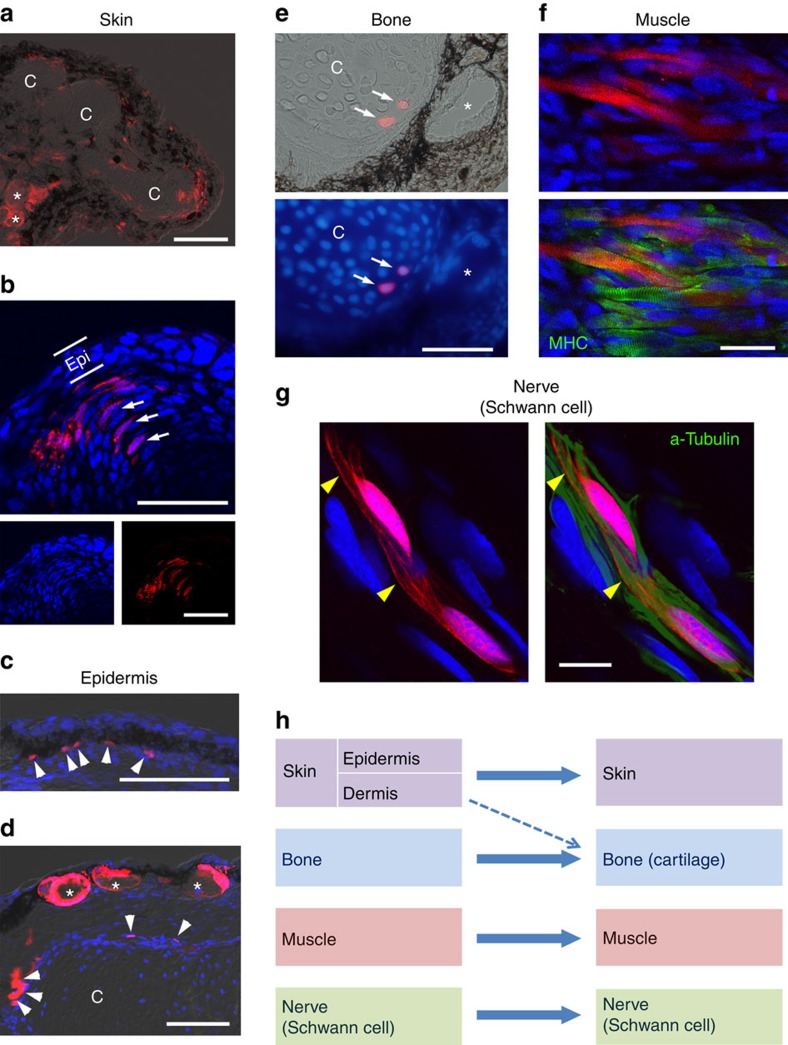
Lineage tracing in adult newt limb regeneration by tissue transplantation. (**a**,**b**) Tracing of skin cells by allograft of mCherry+ skin (*n*=3). (**a**) mCherry+ cells were distributed along the epidermis and dermis, as well as around the cartilage in a regenerating part of the limb (day 40). *c*, cartilage. Asterisk, mucous gland. Scale bar, 200 μm. (**b**) In a growing tip of new digit, mCherry was sometimes observed in chondrocytes (arrows) in the cartilage and in interstitial cells surrounding the cartilage. Epi: epidermis. Scale bars, 100 μm. (**c**,**d**) Tracing of epidermal cells by transplantation of mCherry+ ectoderm at embryonic stage (*n*=2). mCherry was observed in epidermal cells such as those in multilayered epithelium (arrowheads in **c**) and mucous glands (asterisks in **d**), as well as in interstitial cells around the cartilage (arrowheads in **d**) in a regenerating part of the limb (day 148). Scale bars, 200 μm. (**e**) Tracing of bone cells by allograft of mCherry+ bone (*n*=4). mCherry was observed in chondrocytes (arrows) in new cartilage of a regenerating limb (day 81). Asterisk: mucous gland. Scale bar, 100 μm. (**f**) Tracing of muscle cells by allograft of mCherry+ muscle (*n*=3). mCherry was observed in muscle fibres in a regenerating part of the limb (day 81). MHC, myosin heavy chain immunoreactivity. Scale bar, 50 μm. (**g**) Tracing of Schwann cells by implantation of a fragment of mCherry+ nerve (*n*=3). mCherry was observed in Schwann cells along regenerated nerve fibres in a regenerating part of the limb (day 133). Arrowhead: myelin sheath covering a regenerated nerve fibre; a-tubulin, acetylated tubulin immunoreactivity. Scale bar, 20 μm. Blue: nuclei stained by To-pro-3 (**b**–**d**,**f**,**g**) and DAPI (4,6-diamidino-2-phenylindole) (**e**). (**h**) Summary. The skin, bone, muscle and nerve (Schwann cell) regenerated themselves. Exceptionally, dermis contributed to new bone/cartilage.

In axolotl limb regeneration, principal tissues of the limb give rise to the same type of tissues in a lineage-restricted manner. However, the question was left open whether dermis contributes to the cartilage/bones and perhaps vice versa^6^. To examine whether the same is true in the adult newt, we carried out lineage-tracing experiments by transplanting reporter-expressing tissues (Fig. 4 and Supplementary Figs 3–7). In the skin allograft (*n*=3), the skin contributed to a new skin comprising epidermal tissues such as the stratified epithelium, mucous glands and dermal tissues, and also gave rise to interstitial cells, which were mostly distributed around the cartilage in the regenerated limb (Fig. 4a). On rare occasions (in three serial sections from one particular animal), we found a small number of reporter+ chondrocytes in the digit's cartilage (Fig. 4b). In animals into which the reporter+ ectoderm of a presumptive limb was transplanted at its embryonic stage (*n*=2), the epidermis gave rise to a number of interstitial cells and to new epidermal tissues (Fig. 4c,d), but not to cartilage. Therefore, the origin of cartilage might as well be dermal cells as suggested in the axolotl^6^. In the bone allograft experiment (*n*=4), bone contributed to cartilage/bones (Fig. 4e), but did not contribute to other tissues. Reporter+ chondrocytes were observed in almost all sections containing cartilage (1–20 cells per section). In the muscle allograft (*n*=3), as anticipated, the muscle contributed to only the muscle (Fig. 4f). On rare occasions (in two sections from one particular animal), we found a small number of reporter+ chondrocytes in regenerated cartilage. In this case, the grafted tissues may have contained a type of cell that contributed to the cartilage; thus, we regard this result as inconclusive. In the nerve (that is, Schwann cells) implantation (*n*=3), Schwann cells contributed to reconstruction of the myelin sheath that covered a regenerated nerve fibre (Fig. 4g).

Consequently, in terms of tissue origin, the overall design of limb regeneration seemed to be conserved between the adult newt and the axolotl: the principal tissues in the limb strictly regenerated the same tissue types (Fig. 4h), with a possible exception of dermis contributing to the bone/cartilage.

## Discussion

This study provides evidence that the newt is capable of recruiting both Pax7+ MPCs, such as satellite cells, and SMFCs, to restore muscle during limb regeneration. However, the former was the source in the larval stage and the later in the adult stage. In other words, a stem/progenitor-based mechanism is taken over by a dedifferentiation-based one as development proceeds beyond metamorphosis. Therefore, differences in the origin of new muscle between the newt and axolotl should not be attributed to differences in species but rather explained by differences related to developmental processes. It remains to be studied whether the switching of cell source takes place abruptly or gradually after metamorphosis. It must be noted here that lineage tracing of Pax7+ satellite cells cannot be achieved at this point with the technical limitations in newts. The genome of the newt is not sequenced yet and the Pax7 locus is not available; thus, production of transgenic newts is not possible at this point. The axolotl displays neoteny, heterochronically preserving its larval properties even after sexual maturity. Interestingly, in metamorphosed axolotls, satellite cells still contribute to the blastema but there is no SMFCs contribution^2^, and this could be viewed either as a difference among species, as a difference in time after metamorphosis (in axolotls, maybe <10 months) or as a result from a virtual condition for metamorphosis (hormone injection), which may have been insufficient for the switching of cell source or inhibited it. Importantly, our results clearly indicate a degree of plasticity that urolele amphibians could exhibit, to achieve regeneration. It is interesting to note here that in a recent paper it was demonstrated that although preaxial dominance is characteristic of limb regeneration in the larval newt, it changes after metamorphosis with anterior and posterior digits forming together^7^. This is another example of plasticity in limb regeneration mechanism depending on metamorphosis.

Most amphibians studied thus far can regenerate limbs in the larval stage, but after metamorphosis this ability deteriorates or is lost^1^. This is true for the axolotl whose metamorphosis can be induced by thyroid hormone^8^. In contrast, the newt can regenerate limbs regardless of metamorphosis. In general, for animals whose sources of cells for tissue repair and regeneration (for example, stem/progenitor cells and premature cells, which still have potency to change their fate) decrease in number and/or activity during development, it would be advantageous to have the ability to recruit comparable cells through dedifferentiation or reprogramming of mature cells. In amphibians, stem/progenitor cells for muscle regeneration may decrease or deteriorate, or specialize to muscle growth/repair during metamorphosis when the body's system is drastically remodelled to adapt to a terrestrial environment. In fact, in adult newt limbs, the ratio of satellite cells to SMFC nuclei decreases (mean± s.d.: larvae, 14.7±4.9%, *n*=8; adult, 9.3±4.8%, *n*=5; Student's *t*-test, *P*=0.039). However, plenty of satellite cells are still present in the muscle. Therefore, the property of satellite cells and/or surroundings of them that allow them to mobilize into the blastema must change during or after metamorphosis. In a frog (*Xenopus laevis*), it has been suggested that growth factors such as hepatocyte growth factor, which recruit satellite cells into the blastema may decrease during metamorphosis^9^. It may be valuable to examine whether this is true for metamorphosed newts. Possibly, dedifferentiation of SMFCs evolved to enable SMFCs to regenerate the muscle more efficiently in such conditions of adults. In a recent paper^10^, it was shown that newts have the ability to clear senescent cells, so that they do not interfere with regeneration. This mechanism could account for the ability of the newt to regenerate many times at old ages as well^11^. It should also be noted that generation of progenitor cells by dedifferentiation of muscle fibres during adult newt limb regeneration could be attributed to programmed cell death. However, it will be interesting to see whether that is the case in larvae limb regeneration as well^12^.

In summary, both newts and axolotls seem to have the same basic master plan for limb regeneration: tissues in the regenerating limb originate from the parental ones. However, when it comes to muscle, the newt switches from stem/progenitor cells to SMFC dedifferentiation as it transits from metamorphosis. This could bear significance for the loss of regenerative ability in metamorphosed amphibians, such as frogs and other urodele species. Frogs lose their ability for limb and lens regeneration after metamorphosis^13^. Axolotls also lose their ability for lens regeneration as they develop^14^. Perhaps different animals invent new strategies or modify existing ones to suit their regenerative needs. It is interesting to speculate here that the newt overcame loss of limb regeneration after metamorphosis by resorting in part to dedifferentiation. Delineating the mechanisms of these strategies will undoubtedly provide clues for regeneration in other species including mammals.

## Methods

All experiments were carried out in accordance with the guidelines approved by the University of Tsukuba Animal Use and Care Committee.

### Animals

The Japanese fire-bellied newt *C. pyrrhogaster* was used for this study. Fertilized eggs for the transgenic study were obtained from adult Toride-Imori (total body length: male, ∼9 cm; female, 11–12 cm)^3^. Animals were reared at 18 °C under a natural light condition^3^. Developmental stages were determined according to previous criteria^3^. For the tissue transplantation study, adult newts (captured from Okayama and Miyagi Prefecture) purchased from a supplier (Aqua Grace, Yokohama, Japan) were also used as the recipients, unless otherwise noted.

### Anaesthesia

An anaesthetic FA100 (4-allyl-2-methoxyphenol; DS Pharma Animal Health, Osaka, Japan) dissolved in water was used at room temperature (22 °C). Before limb amputation or operations for tissue transplantation, larval (St 56–57; ∼3 months old) and adult newts (∼16 months old and older than 3 years) were anaesthetized in 0.05% FA100 for 15 min, in 0.05% FA100 for 1 h and in 0.1% FA100 for 2 h, respectively.

### Limb amputation

After having been anaesthetized, animals were gently rinsed in distilled water and lightly dried on a paper towel, and one side of the forelimbs of each animal was amputated under a dissecting microscope (M165 FC; Leica Microsystems, Wetzlar, Germany) by a blade (for larvae, a tip of the blade (catalogue number: 4991482, Feather Safety Razor, Osaka, Japan); for adults ∼16 months old and older than 3 years, a surgical blade (No. 14, Futaba, Tokyo, Japan) and microtome blade (C35, Feather Safety Razor), respectively). Next, the larval amputees were allowed to recover in 1 × penicillin–streptomycin (15140–122; Thermo Fisher Scientific, Yokohama, Japan) containing 0.1 × modified Holtfreter's solution^3^ at 22 °C and then reared in the same condition; adult amputees were placed in a moist container and allowed to recover at 14 °C for ∼15 h, and then reared in the same container at 18–20 °C.

### Transgenesis for SMFC tracking

To track SMFCs by means of transgenesis, we constructed a plasmid vector, pCreER^T2^<CarA-CAGGs>[EGFP]mCherry (I-SceI), which carries a transgene cassette flanked by I-SceI meganuclease recognition sites (Supplementary Note 1 and Supplementary Figs 8–12), using conventional molecular cloning procedures. This cassette comprises two expression constructs in opposite directions: one (CAGGs>[EGFP]mCherry) is a reporter construct by which EGFP, whose gene is flanked by loxP sites and followed by the mCherry gene, can be expressed under the control of a universal promoter (CAGGs)^3^; the other (CreER^T2^<CarA) was designed so that an inactive form of Cre recombinase (Cre-ER^T2^: Cre fused to a mutated human oestrogen receptor (ER^T2^))^5^ can be expressed under the control of a cardiac actin promoter (CarA; from M11 Cardiac Actin Promoter pCarA, Addgene 17148), which is known to be activated in both SMFCs and cardiac muscle fibre cells^15^. Each construct is flanked by chicken β-globin HS4 2 × core insulators (kindly provided from Dr Gary Felsenfeld at the National Institute of Health, Bethesda, MD, USA), to minimize possible *cis* interactions within the cassette and between the constructs and functional elements on the chromosome.

We prepared transgenic newts by the I-SceI protocol^3^: one-cell-stage embryos were injected with a construct/enzyme mixture (DNA construct, 80 ng μl^−1^; I-SceI (catalogue number: R06945; New England Biolabs, Tokyo, Japan), 0.5 U μl^−1^; I-SceI buffer (New England Biolabs), 1 × ; Phenol red, 0.01%) at 1–2 μl per embryo and reared until a juvenile stage beyond metamorphosis. In this study, we used swimming larvae (St 56–57) that had developed forelimbs with full digits (total body length: ∼18 mm, age: 3 months; Fig. 1a) and metamorphosed juveniles (total body length: ∼6 cm, age: 16 months; Fig. 3a) (Supplementary Note 1 and Supplementary Fig. 10).

For the study of larval limb regeneration (Figs 1 and 2), we selected swimming larvae that expressed EGFP in their whole body almost evenly and treated them at St 47–53 (age: 1–2 months) in a tamoxifen-containing solution (1–3 μM (Z)-4-hydroxytamoxifen (H7904-5MG, Sigma-Aldrich, St Louis, MO; master mix: 100–300 μM in dimethylsulphoxide) in rearing solution (0.1 × Holtfreter's solution)^3^) for a few weeks (Supplementary Note 1 and Supplementary Figs 10 and 11a). For the study of juvenile limb regeneration (Fig. 3), we used mosaic animals, which expressed EGFP in the muscle only, as well as non-mosaic ones (Supplementary Note 1 and Supplementary Fig. 11b,c). Such mosaic animals were selected at the swimming larval stage. The reason that we used these mosaic animals is to preclude possible contamination of other kinds of cells in SMFC lineage tracing.

In swimming larvae, we carried out amputation in the middle of the forearm. In juveniles, we carried out the first amputation in the middle of the forearm; after the blastema had formed (30–40 days after amputation), we carried out the second amputation in the middle of the upper arm of the same limb, to collect the blastema for histological analysis. These animals were kept for longer than 3 months, to examine later stages of regenerating limbs. The primary reason that we adopted a second amputation on the upper arm is that if we amputated on the forearm, it would be difficult to prove that mCherry+ SMFCs participate in muscle regeneration via dedifferentiation, because similar results can be presumed even when the cells originating from other tissues fuse to the remnant mCherry+ SMFCs in the stump. Amputation in the middle of the upper arm ensures discontinuity between muscles in the regenerated forearm and those in the stump of the upper arm. The secondary reason is that animals to be used, which must be mosaic (see above), were limited.

### Transplantation of reporter-expressing tissues

*CAGGs>reporter* transgenic newts of 1–2 months after metamorphosis were used as donors (Supplementary Figs 13 and 14). The tissues that exhibited intense mCherry/EGFP fluorescence uniformly were transplanted into a wild-type animal (recipient) by fine scissors, pins and forceps under a fluorescent dissecting microscope (M165 FC; Leica). The skin was grafted to a recipient of the same age (Supplementary Fig. 3). The muscle and bone were transplanted individually into the corresponding regions of different recipients 3 years old (Supplementary Figs 5 and 6). The nerve (Schwann cells) was implanted in between muscles of a forelimb of a 3-year-old recipient (Supplementary Fig. 7). In these recipients, after the wound was closed (0.5–1 month after surgery), the limb was amputated so that the grafted tissue remained in the stump. In this study, to track the epidermis, we also transplanted the mCherry+ ectoderm on the presumptive forelimb of a tail bud embryo (St 24–26) to a wild-type embryo at the same stage (Supplementary Fig. 4). In this case, when the recipient reached 6–9 months of age, the forelimb was amputated.

### Microscopic observation of living newts

EGFP/mCherry fluorescence of tissues in living animals was monitored during development, limb regeneration and tissue transplantation under a dissecting microscope (M165 FC; Leica). Specific filter sets for EGFP (Leica GFP-Plant; Exciter: 470/40 nm; Emitter: 525/50 nml) and mCherry (Exciter: XF1044, 575DF25; Emitter: XF3402, 645OM75; Opto Science, Tokyo, Japan) were applied to avoid bleed-through artefacts. Images were taken by a digital camera (C-5060; Olympus, Shinjuku, Tokyo, Japan) attached to the microscope and stored in personal computers.

### Tissue preparation

Larval and adult tissues were fixed in 4% paraformaldehyde in PBS (pH 7.5) at 4 °C for 4–6 and for 15 h, respectively, washed thoroughly with PBS at 4 °C (for larvae, 5 min × 2, 10 min × 2, 15 min × 2, 30 min × 2 and 1 h × 2; for adults, 15 min × 2, 30 min × 2, 1 h × 2 and 2 h) and then allowed to soak in 30% sucrose in PBS at 4 °C. The fixed tissues were embedded into Tissue-Tek O.C.T. Compound (4583; Sakura Finetek USA, Inc., Torrance, CA 90501, USA), frozen at approximately −20 °C in a cryostat (CM1860; Leica) and sectioned at 20 μm thickness. The sections were stored at −20 °C until use. We obtained ∼30, 50–60 and 120–140 dorsoventral sections from larval, juvenile and adult limbs, respectively. For Pax7 immunohistochemistry, larval limbs were fixed in 2% paraformaldehyde/0.2% picric acid in PBS for 1.5 h at 4 °C and sections were obtained in the same way; adult limbs were embedded into Tissue-Tek O.C.T. Compound, immediately frozen in the cryostat, sectioned at 20 μm thickness, fixed in methanol/acetone (1:1; methanol: 134–14,523; acetone: 012–00343; Wako Pure Chemical Industries, Osaka, Japan) at 22 °C for 5 min, air dried for 1–3 h and then stored at −20 °C until use.

### Immunohistochemistry

Immunofluorescence and immunoperoxidase labelling of tissues were carried out^16^. Primary antibodies were as follows: mouse monoclonal anti-Pax7 antibody (1:200; PAX7-c; Developmental Studies Hybridoma Bank, Iowa, IA 52,242–1,324, USA), anti-myosin heavy chain antibody (1:200–336; MF20-c; Developmental Studies Hybridoma Bank), anti-acetylated tubulin antibody (1:1,000; T6793; Sigma-Aldrich, 63103) and rabbit polyclonal anti-collagen type IV antibody (1:500; Code: 600–401–106–0.1; Rockland Immunochemicals, Gilbertsville, PA 19525, USA). Secondary antibodies were as follows: Alexa Fluor 488-conjugated goat anti-mouse IgG (H+L) (1:500; A11001; Thermo Fisher Scientific), biotinylated goat anti-mouse IgG (1:400; BA-9200; Vector Laboratories, Burlingame, CA 94010, USA) and rhodamine (TRITC)-conjugated affiniPure goat anti-rabbit IgG (H+L) (1:500; Code: 111-025-003; Jackson ImmunoResearch Laboratories, West Grove, PA 19390, USA). For immunofluorescence labelling, tissue sections were rinsed thoroughly (PBS, 0.2% Triton X-100 in PBS and PBS; 15 min each), incubated in a blocking solution (3% normal goat serum (S-1000, Vector Laboratories)/0.2% Triton X-100 in PBS) for 2 h, washed twice in PBS and then incubated in primary antibody diluted with blocking solution for 15 h at 4 °C. After washing thoroughly, the samples were incubated in secondary antibody diluted with blocking solution for 4 h and washed thoroughly. For immunoperoxidase labelling, thoroughly rinsed tissues were treated with 3.3% H_2_O_2_ in methanol for 20 min, washed thoroughly, incubated in a blocking solution containing Avidin D (1:50; Avidin/Biotin Blocking kit, SP-2001, Vector Laboratories) for 2 h, washed twice in PBS and then incubated in primary antibody diluted with blocking solution containing Biotin (1:50; Avidin/Biotin Blocking kit) for 15 h at 4 °C. After washing thoroughly, the samples were incubated in biotinylated secondary antibody diluted with blocking solution for 4 h, washed thoroughly, incubated in a mixture of Avidin and Biotin Complex (Vectastain ABC Elite kit, PK-6100, Vector Laboratories) for 2 h, washed thoroughly and then incubated in a DAB solution (DAB substrate kit, SK-4100; Vector Laboratories). In both labelling, after the samples were washed, the nuclei of cells were counterstained with 4,6-diamidino-2-phenylindole (1:50,000; D1306; Thermo Fisher Scientific) or TO-PRO-3 Iodide (1:50,000; T3605; Thermo Fisher Scientific).The tissues were immersed into 90% glycerol in PBS or into VECTASHIELD mounting medium (H-1000; Vector Laboratories) and then mounted under a cover slip.

### Image analysis

Images of tissues were acquired either by a charge-coupled device camera system (DP73; cellSens Standard 1.6; Olympus) attached to a microscope (BX50; Olympus) or through a confocal microscope system (LSM510; LSM 5.0 Image Browser software; Carl Zeiss, Germany). Images were analysed by Photoshop CS5 Extended (Adobe Systems, San Jose, CA) and with software for the image acquisition systems. Figures were prepared using Photoshop CS5 Extended. Image, brightness, contrast and sharpness were adjusted according to the journal's guidelines.

## Additional information

**How to cite this article:** Tanaka, H. V. *et al.* A developmentally regulated switch from stem cells to dedifferentiation for limb muscle regeneration in newts. *Nat. Commun.* 7:11069 doi: 10.1038/ncomms11069 (2016).

## Supplementary Material

Supplementary Information Supplementary Figures 1-14, Supplementary Note 1 and Supplementary References.

Supplementary Movie 1 Monitoring of SMFCs (mCherry+) during larval newt limb regeneration. See Fig. 1b for details.

Supplementary Movie 2 Fragments of muscle fibers near the stump and mononucleated cells in the blastema in a day 36 regenerating limb of juvenile newt. See Supplementary Figure 2 for details.

## Figures and Tables

**Table 1:** Appearance of mCherry+ mononucleated cells during larval limb regeneration.

**Before amputation**	**Blastema**	**Late regenerating limb**
0/40 (0%)	0/15 (0%)	9/9 (100%)

The value is the ratio of animals in which we observed mCherry+ mononucleated cells either in the body before amputation, in the blastema or in the late regenerating limb.
