# Supplementary material for: A developmentally regulated switch from stem cells to dedifferentiation for limb muscle regeneration in newts
Source: Nat Commun. 2016 Mar 30;7:11069. doi: 10.1038/ncomms11069 (PMC4820895; doi:10.1038/ncomms11069)
Supplement: Supplementary Information — Supplementary Figures 1-14, Supplementary Note 1 and Supplementary References. [file ncomms11069-s1.pdf]

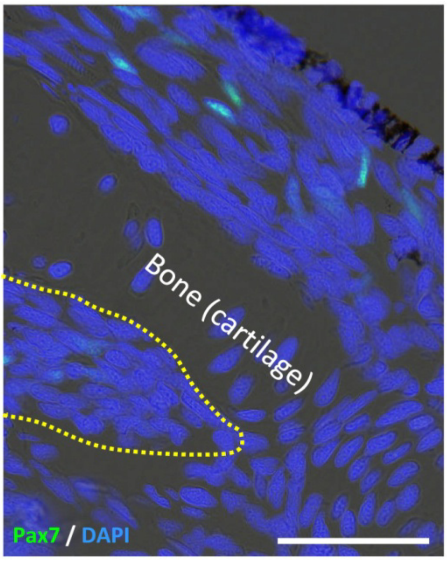

Supplementary Figure 1 | Mononucleated cells in the mCherry positive flexor area (as described in Fig. 2) are Pax7 negative (dotted line). In this image, a few Pax7 positive nuclei are recognized in the same area. These cells are probably satellite cells which have migrated from the stump as described in Fig. 1. Scale bar: 100 µm.

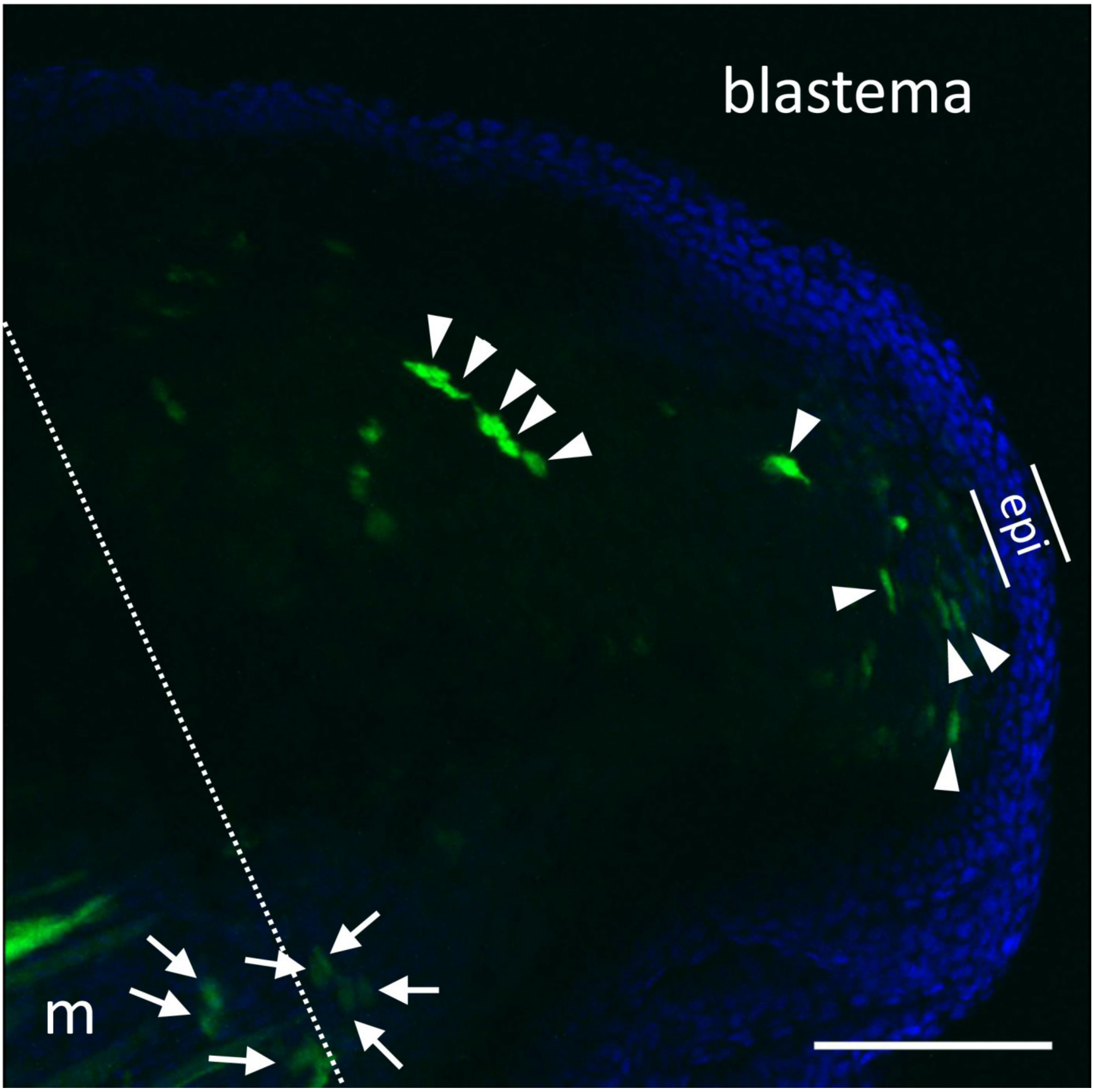

Supplementary Figure 2 | A confocal image of a section that was obtained from the same regenerating juvenile limb (day 36) shown in Fig. 3c and d. 3D-image of this section is shown in Supplementary Movie 2. In this section, only EGFP+ muscle fiber-derived cells (green) were observed. Fragments of muscle fibers near the stump are indicated by arrows, and mononucleated cells in the blastema are indicated by arrowheads. Dotted line: amputation site. *m*: muscle. *epi*: epidermis. Scale bar: 200 µm.

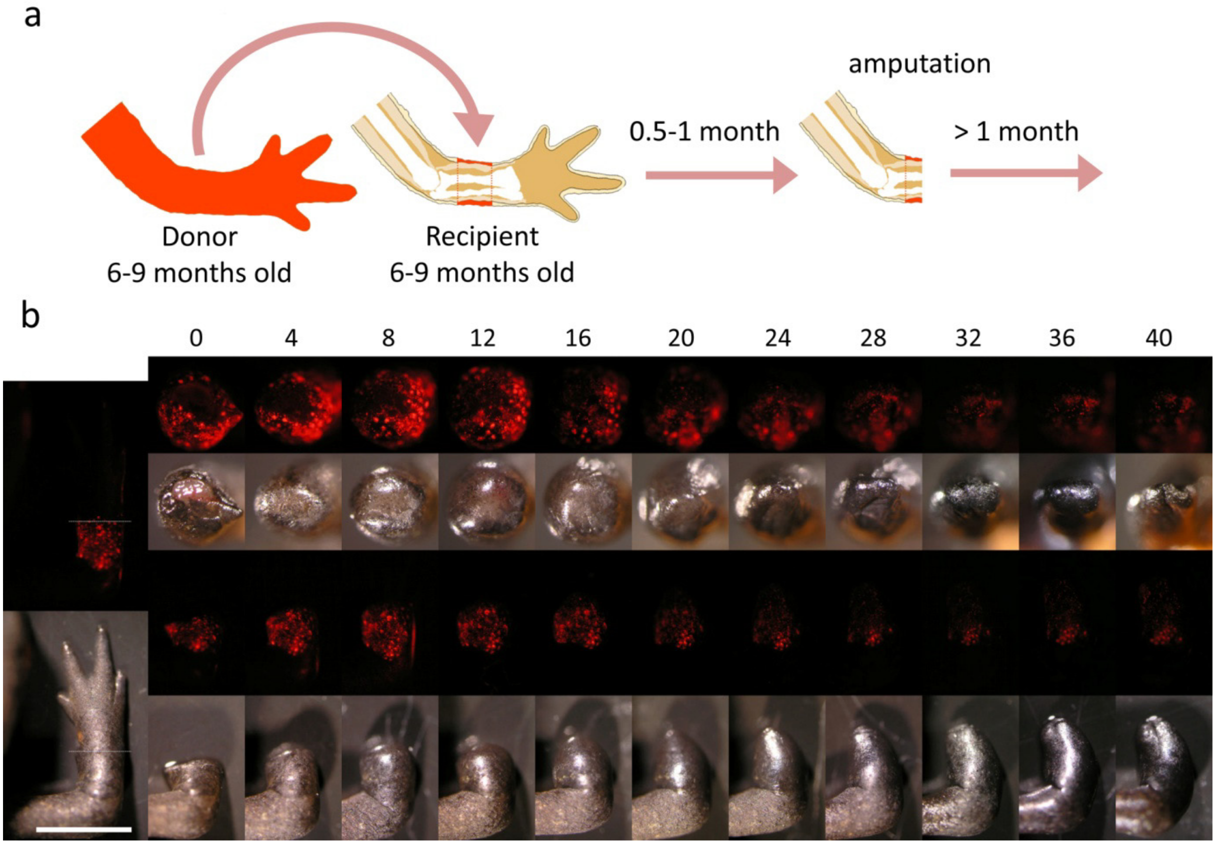

Supplementary Figure 3 | Skin allograft. a, Schematic of experiment. The skin surrounding the forearm of a recipient was replaced with that (mCherry+) of a donor of the same age (6-9 months old), the mCherry+ skin was allowed to integrate to neighboring tissues for 0.5-1 month, and then the limb was amputated along the line across the mCherry+ skin. **b,** Apical and dorsal views of an amputated forelimb. The number in each column indicates days after amputation. In this case, the wound was closed by an epithelium with high transparency (i.e., wound epidermis) within 4 days after amputation. In 12 days, the wound epidermis was covered by another highly fluorescent epithelial sheet that contained pigments and the mucous glands. As regeneration proceeded, fluorescence of the new skin declined while its pigmentation increased. Note that most mucous glands were left around the amputation site. Tissue sections of the regenerating limb of this animal on Day 40 are shown in Fig. 4a and b. Scale bars: 2.5 mm.

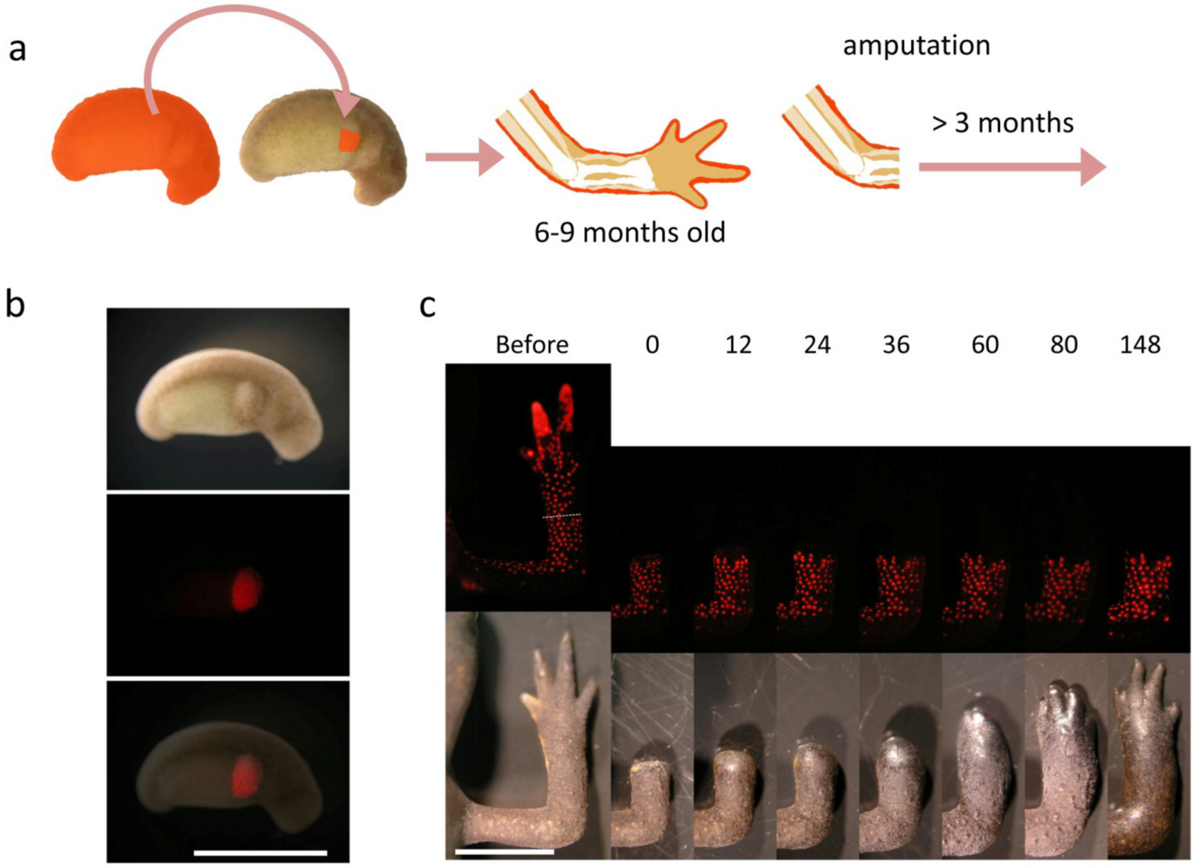

Supplementary Figure 4 | Ectoderm transplantation. a, Schematic of experiment. The mCherry+ ectoderm on the presumptive forelimb of a tail bud embryo (St. 24-26) was transplanted to a wild type embryo at the same stage as described previously[1]. A sample embryo after transplantation is shown in **b**. When the recipient reached 6-9 months of age, the forelimb was amputated. **c,** Dorsal view of a regenerating limb. The number on each column indicates days after amputation. mCherry fluorescence, which was observed throughout the surface of the intact forelimb (except in the region covered by pigmented cells), was obscure in regenerating part of the limb. This was partly due to pigmentation in regenerating skin. Note that most mucous glands did not migrate from the amputation site. Tissue sections of the regenerating limb of this animal on Day 148 are shown in Fig. 4c and d. Scale bars: 2.5 mm. Note: In either the skin allograft (Supplementary Figure 3) or ectoderm transplantation (here), tissue sections of regenerated limbs revealed that cells in the new epidermal tissues were not always reporter+ (Fig. 4a-d). Since the CAGGs promoter is not repressed in new epidermal tissues[2], additional cellular sources for skin regeneration may be needed.

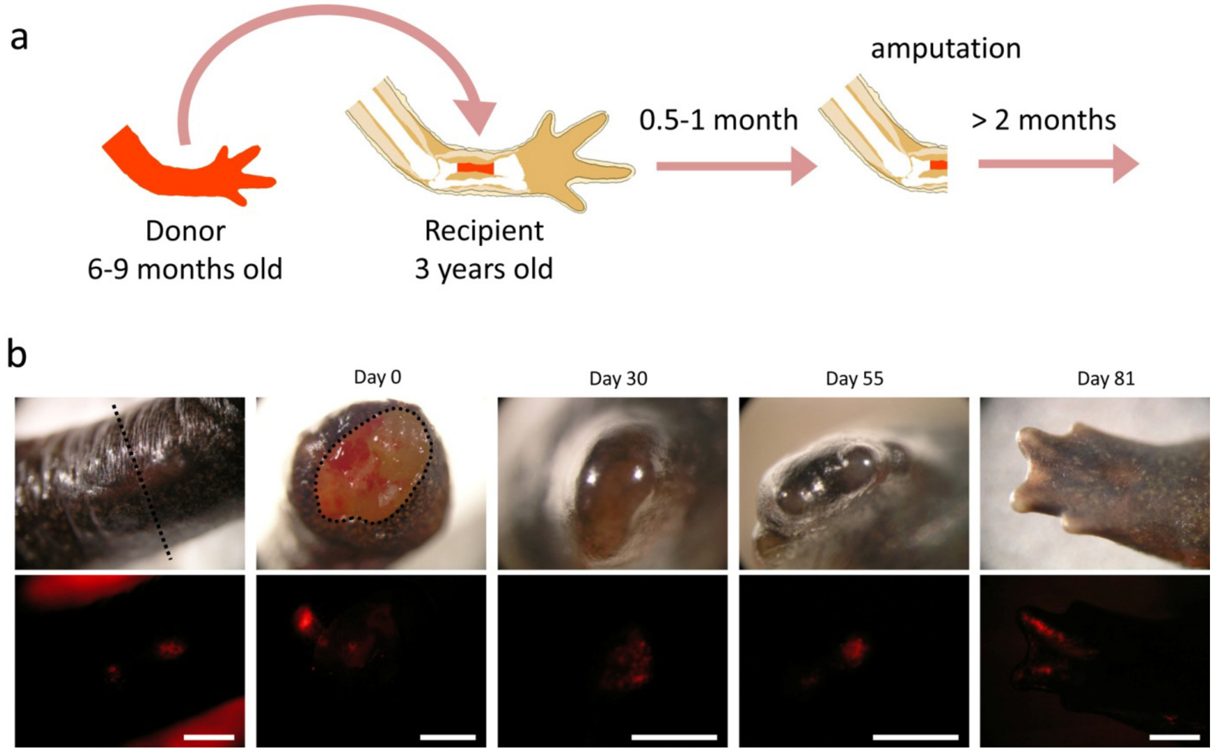

Supplementary Figure 5 | Bone allograft. a, Schematic of experiment. An mCherry+ bone isolated from the forearm of a donor (6-9 months old) was transplanted to the corresponding region of a recipient (3 years old). After the wound was closed (0.5-1 month after surgery), the limb was amputated so that the grafted bone remained in the stump. **b,** Dorsal view of a closed wound from which a mCherry+ bone was transplanted (left hand column), apical views of the same limb immediately (day 0) and on day 30 and 55 after amputation, and a dorsal view on day 81. mCherry fluorescence was recognized through transparent regions of the skin throughout the process of regeneration. A tissue section of the regenerating limb of this animal on Day 81 is shown in Fig. 4e. Scale bars: 1 mm.

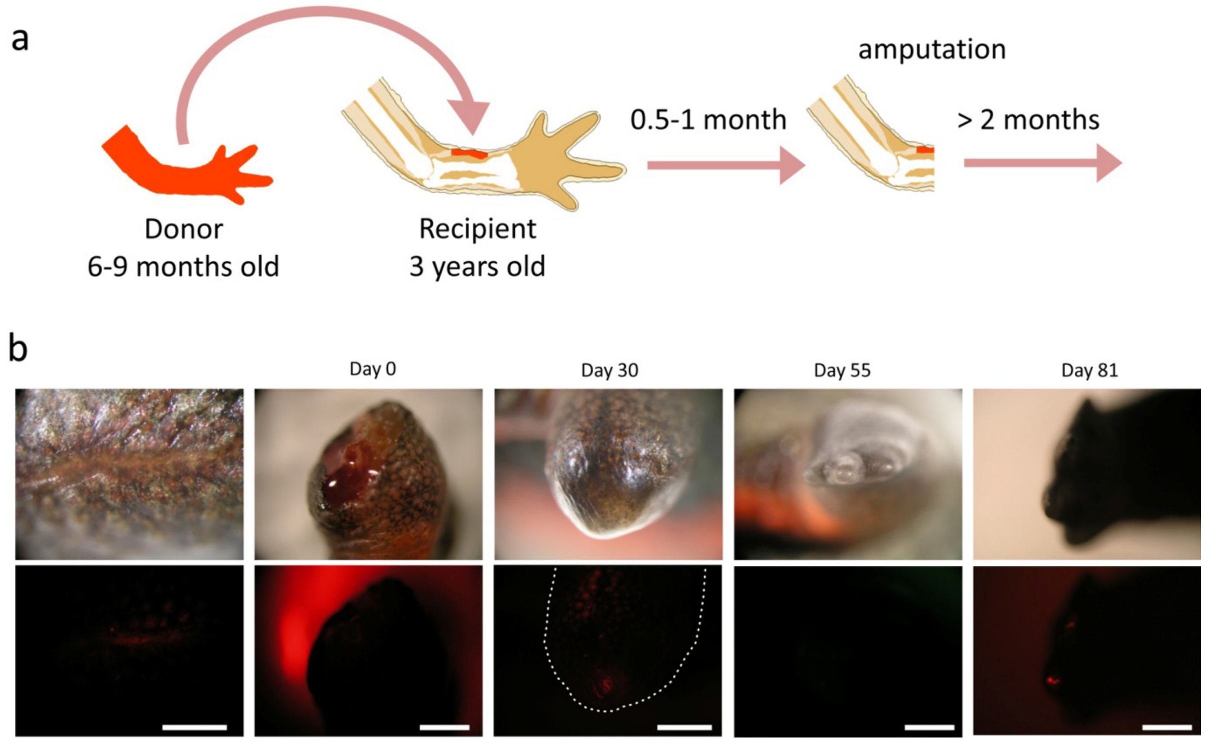

Supplementary Figure 6 | Muscle allograft. a, Schematic of experiment. An mCherry+ muscle isolated from the forearm of a donor (6-9 months old) was transplanted to the corresponding region of a recipient (3 years old). After the wound was closed (0.5-1 month after surgery), the limb was amputated so that the grafted muscle remained in the stump. **b,** Dorsal view of a closed wound from which an mCherry+ muscle was transplanted (left hand column) and apical views of the same limb immediately (Day 0) and on Day 30, 55 and 81 after amputation. mCherry fluorescence was recognized through transparent regions of the skin throughout the process of regeneration. A tissue section of the regenerating limb of this animal on Day 81 is shown in Fig. 4f. Scale bars: 1 mm. In some experiments, animals of the same age as donors were also used as recipients.

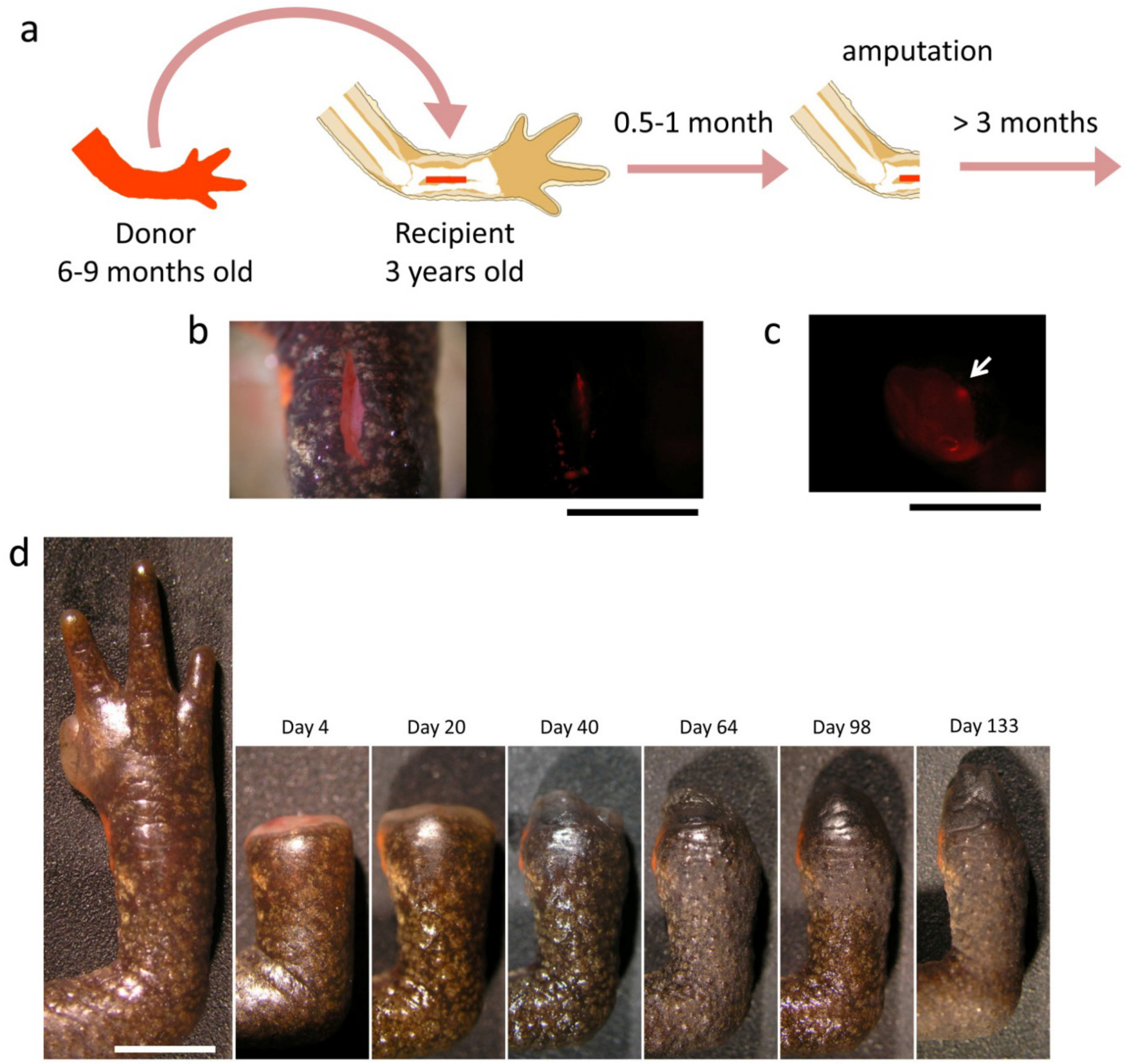

Supplementary Figure 7 | Nerve implantation. a, Schematic of experiment. A fragment of mCherry+ nerve isolated from a forearm of the donor (6-9 months old) was implanted in between muscles in the dorsal side of a forelimb of the recipient (3 years old). After the wound closed (0.5-1 month after surgery), the limb was amputated so that the implanted nerve remained in the stump. **b,** Dorsal view of a wound immediately after implantation of an mCherry+ nerve. **c,** Apical view of the stump on Day 4. The arrow indicates the implanted nerve. **d,** Dorsal views of the same limb during regeneration. In this case, regeneration was slower than in other cases where the implant in the stump could not be recognized. A tissue section of the regenerating limb of this animal on Day 133 is shown in Fig. 4g. Scale bars: 5 mm.

pCreERT2<CarA-CAGGs>[EGFP]mCherry (I-SceI)

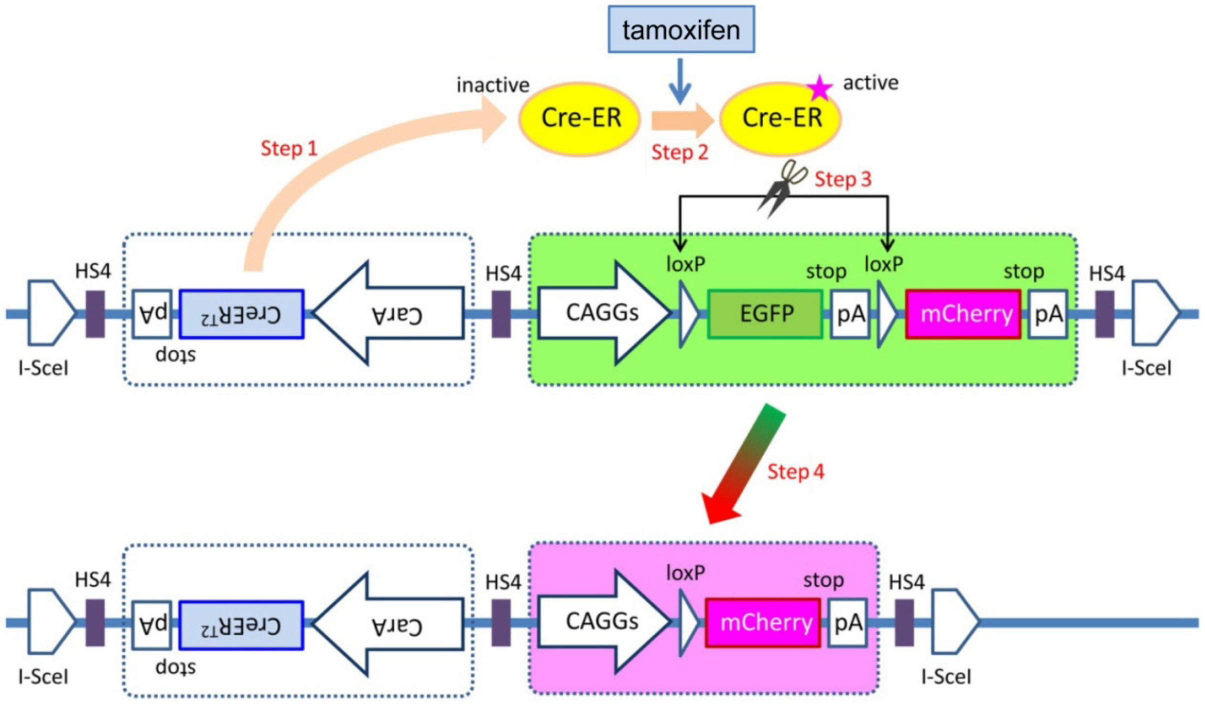

Supplementary Figure 8 | Construct design. We hypothesized that in transgenic newts exhibiting EGFP fluorescence throughout their body, the inactive form of Cre would also be expressed in SMFCs in their limbs (Step 1); when Cre is activated by administration of tamoxifen (Step 2), the *EGFP* gene in the reporter construct is removed and the *mCherr*y gene is ligated to *CAGGs* (Step 3), making the SMFCs express mCherry (Step 4).

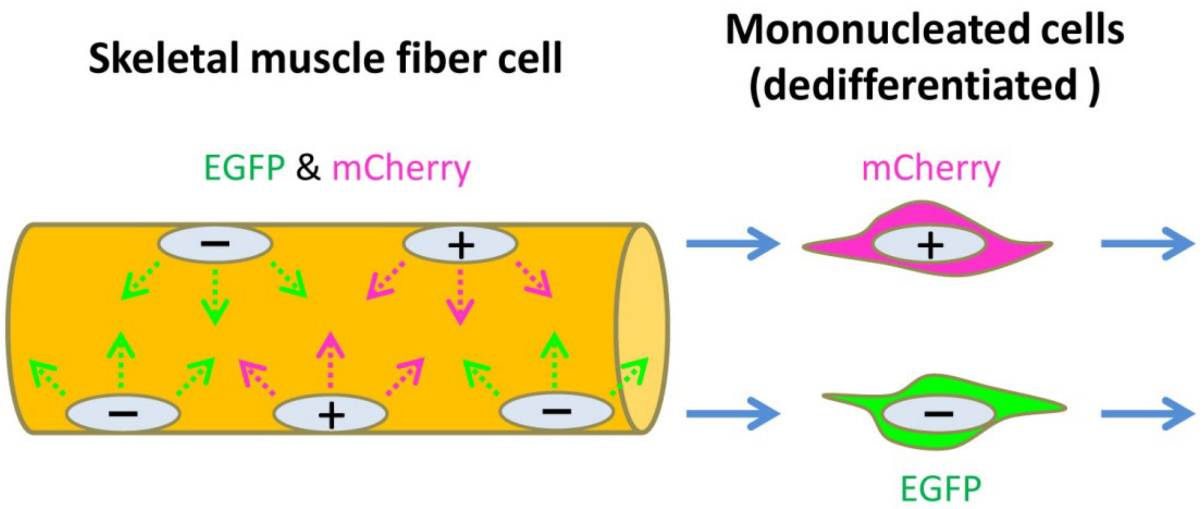

Supplementary Figure 9 | Predicted labeling patterns in SMFC and SMFC-derived mononucleated (dedifferentiated) cells. The SMFC is a syncytium or multinuclear cell. In our labeling system, the cell should express both EGFP and mCherry because of moderate recombination efficiency (see Supplementary Fig. 12), and exhibit fluorescence of both along the fiber. However, as the cell dedifferentiates into mononucleated cells, these cells should exhibit either EGFP or mCherry fluorescence. Plus (+) indicates a nucleus that has been recombined by tamoxifen-activated Cre, and minus (-) indicates a non-recombined nucleus.

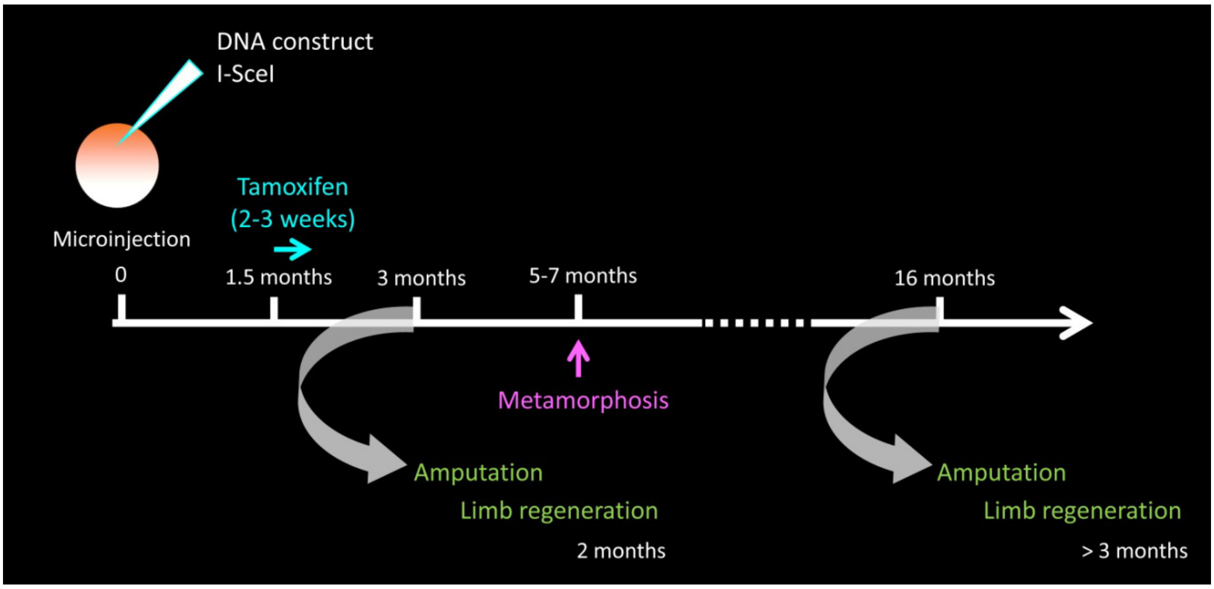

Supplementary Figure 10 | Time schedule of experiments. Fertilized eggs (or one-cell stage embryos) were injected with pCreER^{T2}<CarA-CAGGs>[EGFP]mCherry (I-SceI)/I-SceI enzyme mixture. Swimming larvae at St. 47-53 (age: 1-2 months) were treated in a tamoxifen-containing solution for a few weeks. In this study, we examined swimming larvae (St. 56-57) which had developed forelimbs with full digits (total body length: ~18 mm; age: 3 months; Fig. 1a) and metamorphosed juveniles (total body length: ~6 cm; age: 16 months; Fig. 3a).

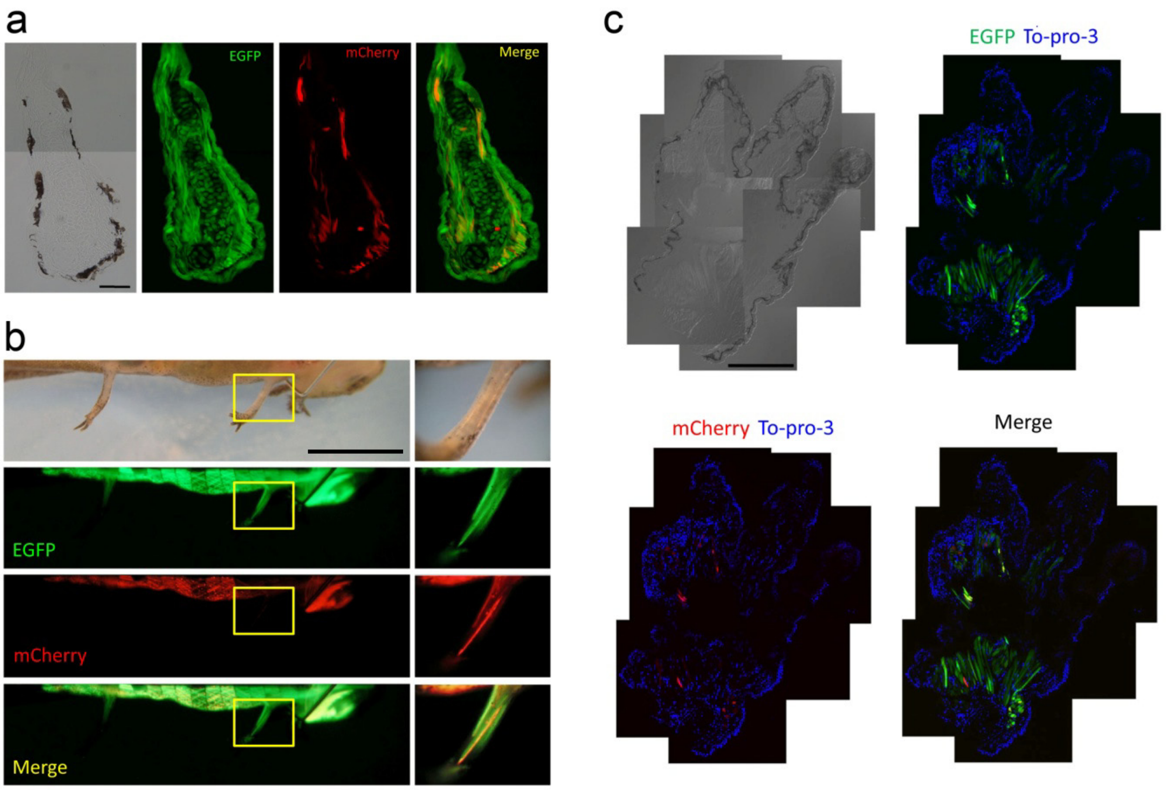

Supplementary Figure 11 | mCherry expression in larval and juvenile muscles. a, A section of the forelimb of a swimming larva which expressed EGFP in its whole body almost uniformly. Scale bar: 100 µm. **b,** Ventral view of another swimming larva. This animal was mosaic and EGFP was expressed exclusively in muscle fibers. Scale bar: 5 mm. Right hand panels are enlargements of the area enclosed by yellow rectangles. In both larvae, almost all of the muscle fibers showed mCherry as well as EGFP fluorescence (**a,b**). **c,** A section of the forelimb (hand) of the animal in **b** at the juvenile stage. Even though the total number of mCherry+ fibers in the limb that had been labeled in the larval stage (St. 47-53) by tamoxifen did not seem to be different between larvae (St. 56-57) and juveniles (16 months), the ratio of mCherry+ fibers in juvenile limb muscle was obviously low compared to that in larval muscle. This is because new fibers (EGFP+/mCherry-) appeared and increased in number in the muscle as the limb grew after metamorphosis. To-pro-3 shows nuclei. Scale bar: 500 µm.

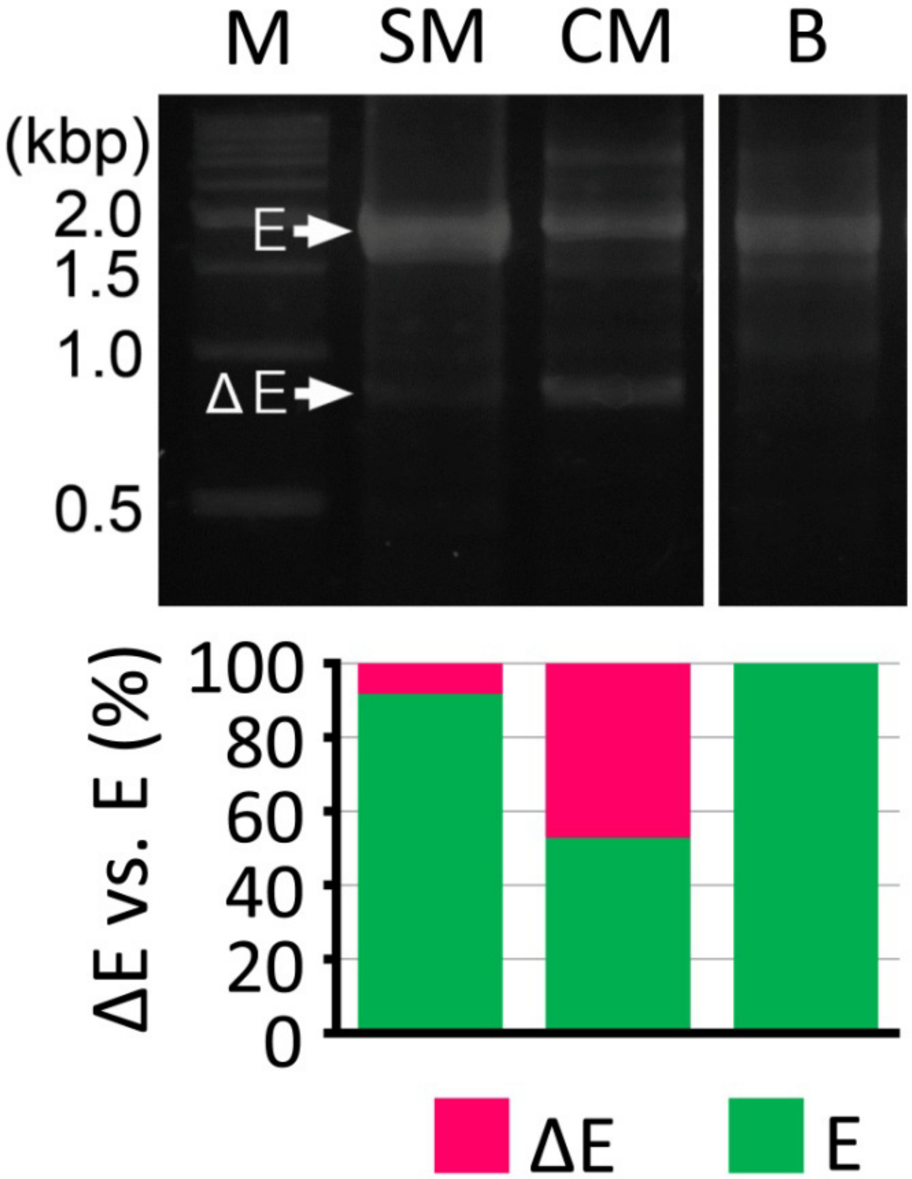

Supplementary Figure 12 | Recombination in the transgene cassette. Upper panel, PCR detection of the transgene cassette with (E; 1,836 bp) or without floxed *EGFP* (ΔE; 841 bp) in genomic DNA. **Lower panel,** Recombination ratio (%) estimated from the relative intensity of PCR bands (ΔE/E). In juvenile newts, the recombination ratio was 8.2% in the skeletal muscle (SM) of the forelimb and about 50% in the cardiac muscle (CM) of the heart. On the other hand, recombination was not detected in the brain (B). M: marker.

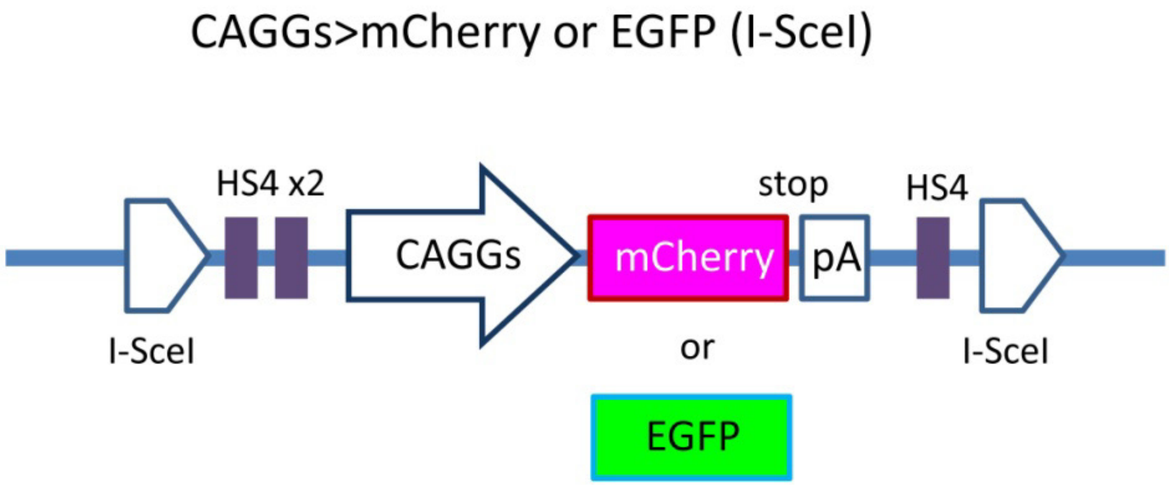

Supplementary Figure 13 | Reporter constructs for cell tracking by tissue transplantation. With this tool, a reporter (mCherry or EGFP) can be expressed universally in the newt under the control of the CAGGs promoter[2].

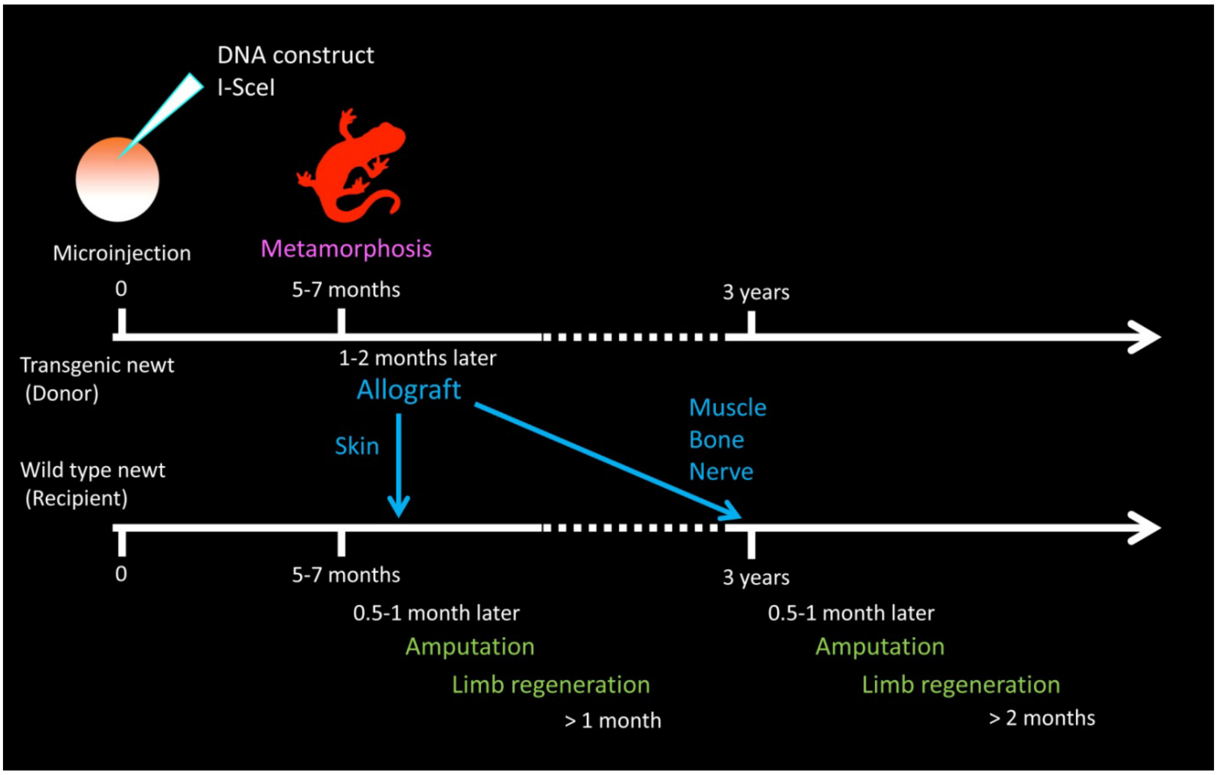

Supplementary Figure 14 | Time schedule of cell tracking by transplantation of reporter expressing tissues. *CAGGs>reporter* transgenic newts of 1-2 months after metamorphosis were used as donors. The skin, muscle, bone and nerve of the forearm, which exhibited intense mCherry/EGFP fluorescence uniformly, were isolated from the donors in a saline solution[3] by fine scissors, pins and forceps under a fluorescent dissecting microscope (Leica M165 FC). The skin was grafted to a wild type animal (recipient) of the same age. The muscle and bone were transplanted individually into the corresponding regions of different recipients 3 years old. The nerve (Schwann cells) was implanted in between muscles of a forelimb of a 3-year-old recipient. Details are illustrated in Supplementary Fig. 3 and 5-7.

Supplementary Note 1. Transgenesis for SMFC tracking

Construct design

A plasmid vector, pCreER^{T2}<CarA-CAGGs>[EGFP]mCherry (I-SceI) was designed to track skeletal muscle fiber cells (SMFCs) by means of transgenesis (Supplementary Fig. 8). If this system works, SMFCs can be labeled before amputation of the limb to identify cells that originate from these cells during limb regeneration because the *CAGGs* promoter is activated regardless of cell type or dedifferentiation. In fact, in the newt, promoter activity is persistent throughout limb regeneration[2].

Predicted labeling patterns

In this system, however, the SMFC (a syncytium or multinuclear cell) after tamoxifen treatment should have both EGFP and mCherry because recombination by tamoxifen is generally known to be imperfect (i.e., not all nuclei undergo recombination; also see Supplementary Fig. 12) and both tracers can diffuse throughout the cytoplasm along the fiber (Supplementary Fig. 9). In fact, in all animals (n=40) we examined in this study, SMFCs exhibiting mCherry fluorescence also had EGFP fluorescence (fibers expressing mCherry only were very rare) (see Supplementary Fig. 11). On the other hand, mononucleated cells (or dedifferentiated cells from muscle fibers) in the blastema exhibited, as predicted in Supplementary Figure 9, either EGFP or mCherry fluorescence (Fig. 3d, Day 36), corroborating the binary regulation of gene expression in this system.

If the mCherry+ mononucleated cells fuse with other EGFP+ cells to regenerate a muscle fiber, the fiber should show both EGFP and mCherry fluorescence, although the intensity of mCherry fluorescence is dependent upon the number of recombined nuclei in the fiber and the volume of the fiber. In fact, as we showed in Fig. 3e (Day 96), regenerated muscle fibers exhibited fluorescence of both but with variable intensity of mCherry fluorescence.

Time schedule of experiments

Transgenic newts to track SMFCs were prepared by the I-SceI protocol[2] (Supplementary Fig. 10). For the study of larval limb regeneration (Fig. 1 and 2), we selected swimming larvae which expressed EGFP in their whole body almost evenly (Supplementary Fig. 11a). For the study of juvenile limb regeneration (Fig. 3), we used mosaic animals, which expressed EGFP in the muscle only (Supplementary Fig. 11b, c), as well as non-mosaic ones.

mCherry expression patterns in larval and juvenile newts

In swimming larvae which exhibited fluorescence of EGFP in their whole body almost evenly, we detected mCherry expression in almost all of the SMFCs in the body (Supplementary Fig. 11a) as well as in the heart (not shown), but not in other body parts. Note that EGFP-/mCherry+ mononucleated cells like those observed in late regenerating limbs (Fig. 2a,b) were never observed in those animals (n=40; Table 1). After metamorphosis, as the

muscle grew, EGFP+/mCherry- striated fibers appeared in the muscle and increased in number. As a result, in juveniles (16 months), EGFP+/mCherry+ fibers became the minority (in the forelimb, only 21-26% of total muscle fibers expressed mCherry; Supplementary Fig. 11c, Fig. 3).

Recombination efficiency

To examine the recombination efficiency of this system, we harvested EGFP+/mCherry+ SMFCs-containing muscles from the forelimbs of juvenile newts instead of those of larvae because the amount of muscles/DNA per animal was very small in the larval stage. We purified genomic DNAs (NucleoSpin® Tissue XS; MACHEREY-NAGEL GmbH & Co., Germany) and carried out PCR with a primer set (forward: 5'-acagctcctgggcaacgtgctggttgt-3'; reverse: 5'-ccggtggagtggcggccctcggcgc-3') designed to amplify a 1,836 bp region containing the floxed *EGFP* sequence (841 bp) in the transgene cassette. PCR results and DNA sequencing revealed that recombination to ligate the *mCherry* gene to the *CAGGs* promoter had taken place in skeletal muscle (Supplementary Fig. 12), although the ratio of the transgene cassettes, which lost their floxed *EGFP* sequence, was low (8.2%). Thus, the recombination efficiency seemed to be low but as for one SMFC this value is an underestimate because 74-79% of the juvenile muscle fibers did not express mCherry (see above; Supplementary Fig. 11c). We should note that in this system recombination had also taken place in the heart (recombination efficiency in cardiac muscle was about 50%), but was not detected in other body parts such as the brain (Supplementary Fig. 12).

Supplementary References

1. Takaya, H. Experimental study on limb-asymmetry. *Zool. Sci.* **20,** 181-279 (1941).

2. Casco-Robles, M. M. *et al.* Expressing exogenous genes in newts by transgenesis. *Nature Protoc.* **6,** 600-608 (2011).

3. Nakamura, K. *et al.* A transcriptome for the study of early processes of retinal regeneration in the adult newt, *Cynops pyrrhogaster*. *PLoS One* 9, e109831 (2014).
